# Rare antibody-associated hemolytic transfusion reaction and transfusion-related acute lung injury: a case report

**DOI:** 10.1186/s12893-017-0241-y

**Published:** 2017-04-26

**Authors:** Tim N. Beck, Natalee G. Young, Michelle L. Erickson, Ignacio Prats

**Affiliations:** 1 Molecular and Cell Biology and Genetics, Drexel University College of Medicine, Philadelphia, PA 19129 USA; 2 Molecular Therapeutics, Fox Chase Cancer Center, Philadelphia, PA 19111 USA; 3 Department of Surgery, WellSpan York Hospital, York, PA 17403 USA; 4 Department of Pathology/Blood Bank, WellSpan York Hospital, York, PA 17403 USA; 5 Leader Surgical Associates, Leader Surgical Center, York, PA 17403 USA

**Keywords:** Hemolytic transfusion reaction, Transfusion-related acute lung injury (TRALI), Thrombocytopenia, Allo-antibodies, Blood products, Direct antiglobulin tests (DAT)

## Abstract

**Background:**

Hemolytic transfusion reactions and transfusion-related acute lung injury (TRALI) are life-threatening complications associated with the transfusion of blood products. Hemorrhage is one of the most common surgical complications and the risk of bleeding is particularly acute in patients with hematologic deficiencies. Management of surgical bleeding can be divided into two phases. The first phase centers on immediate control of acute bleeding and the second phase focuses on keeping the patient stable and on reducing the sequelae associated with blood transfusions and blood loss.

**Case presentation:**

We present the case of a 53-year-old woman with long-standing immune thrombocytopenia who underwent repair of a symptomatic ventral hernia. On post-operative day one the patient developed hemoperitoneum, requiring exploratory laparotomy and massive transfusion of blood products. The patient’s recovery was complicated by consistently low hemoglobin, hematocrit and platelets, prompting frequent transfusion of additional blood products. Shortly after activation of the massive transfusion protocol, the patient developed TRALI. Compounding the situation, on post-operative day sixteen the patient’s serum started to show hemolysis: lactate dehydrogenase (LDH) levels rose to 1,845 IU/L, with haptoglobin at less than 5.8 mg/dL and with a high reticulocyte count (4.38%). Previous testing had shown that the patient was positive for most major antigens implicated in antibody formation and was only producing anti-E and anti-K antibodies (considered for all transfusions). Initial pre- and post-transfusion direct antiglobulin tests (DAT) were indeed negative. However, repeat DATs in the days following the noted serum changes were consistent with new allo-antibody formation. These findings prompted immediate withholding of all blood products and a thorough blood bank work up. Despite strong evidence for new allo-antibody formation, no specific known antibody could be identified. The patient recover well when blood products were withheld.

**Discussion:**

We present the case of a 53-year-old woman with long-standing immune thrombocytopenia who underwent repair of a symptomatic ventral hernia. On post-operative day one the patient developed hemoperitoneum, requiring exploratory laparotomy and massive transfusion of blood products. The patient’s recovery was complicated by consistently low hemoglobin, hematocrit and platelets, prompting frequent transfusion of additional blood products. Shortly after activation of the massive transfusion protocol, the patient developed TRALI. Compounding the situation, on post-operative day sixteen the patient’s serum started to show hemolysis: lactate dehydrogenase (LDH) levels rose to 1,845 IU/L, with haptoglobin at less than 5.8 mg/dL and with a high reticulocyte count (4.38%). Previous testing had shown that the patient was positive for most major antigens implicated in antibody formation and was only producing anti-E and anti-K antibodies (considered for all transfusions). Initial pre- and post-transfusion direct antiglobulin tests (DAT) were indeed negative. However, repeat DATs in the days following the noted serum changes were consistent with new allo-antibody formation. These findings prompted immediate withholding of all blood products and a thorough blood bank work up. Despite strong evidence for new allo-antibody formation, no specific known antibody could be identified. The patient recover well when blood products were withheld. Suspicion for hemolytic transfusion reactions should be high in patients with prior allo-antibody formation; these may present as acute hemolysis or as a delayed hemolytic transfusion reaction. Withholding blood products from these patients until compatible products have been identified is recommended. Moreover, TRALI is the leading cause of transfusion-related fatalities and should always be considered in transfusion settings.

**Conclusions:**

Suspicion for hemolytic transfusion reactions should be high in patients with prior allo-antibody formation; these may present as acute hemolysis or as a delayed hemolytic transfusion reaction. Withholding blood products from these patients until compatible products have been identified is recommended. Moreover, TRALI is the leading cause of transfusion-related fatalities and should always be considered in transfusion settings.

## Background

**Fig. 1:**
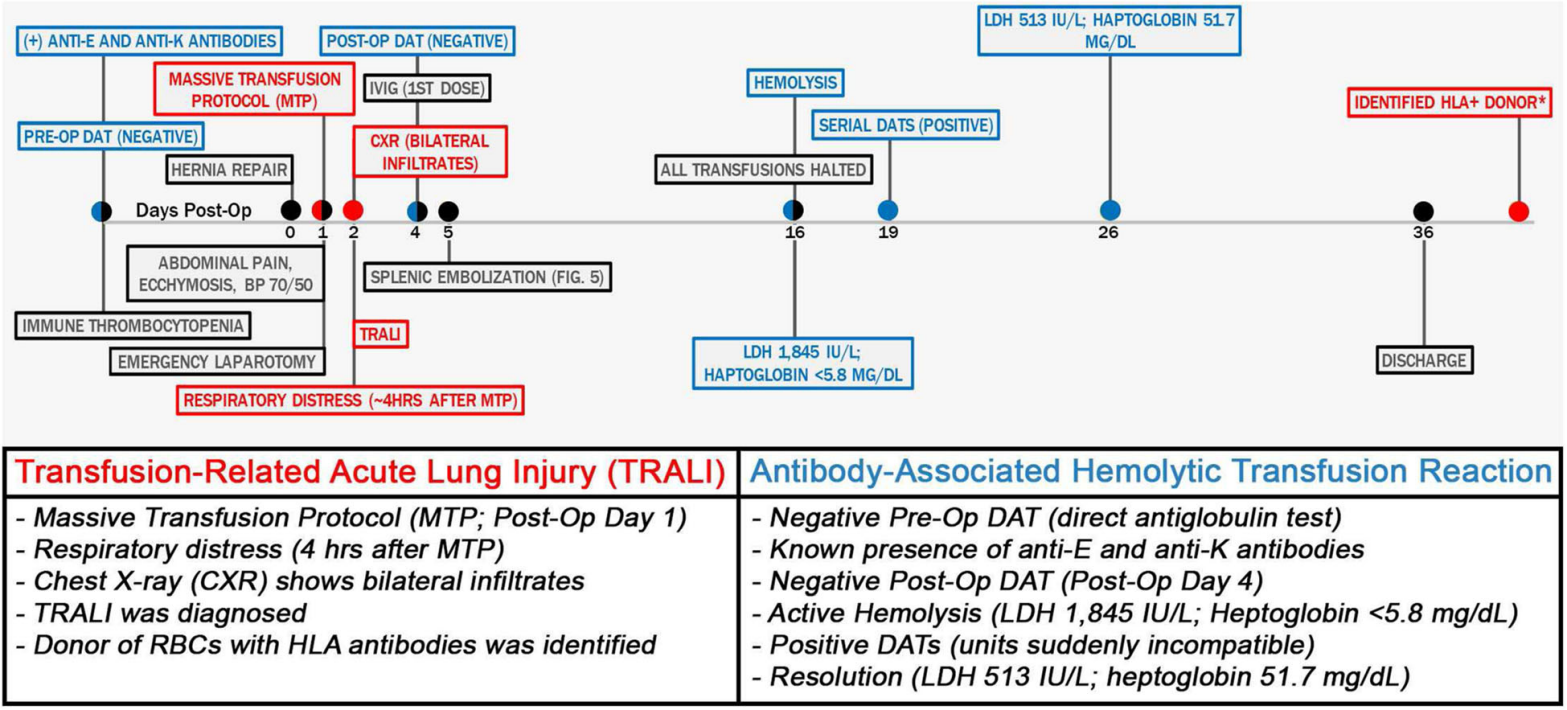
Timeline of pertinent events. BP – blood pressure; HLA+ – positive anti-human leucocyte antigen; LDH - lactate dehydrogenase; Pre-op – pre-operative; Post-op – post-operative; IVIG - Intravenous immunoglobulin; red = related to TRALI; Blue = related to antibody-associated hemolytic hemolytic transfusion reaction. *Multiparous donor tested positive for HLA antibodies

This case report describes the management of post-operative bleeding with focus on adverse blood transfusion associated events. Figure 1 provides a timeline of events pertinent to this case. The aim of this report is to highlight some of the challenges associated with blood transfusions and propose judicious use of blood products. Transfusion associated adverse events should be considered in cases that require activation of a massive transfusion protocol (MTP), frequently defined as transfusion of 10 units of blood or more in a 24-h period [1, 2]. The transfusion of blood products is often lifesaving; however, it does carry a significant risk and care must be taken. Two particularly egregious complications associated with blood transfusions are delayed hemolytic transfusion reactions (DHTR; [3, 4]) and transfusion-related acute lung injury (TRALI; [5, 6]).

Per the US Food and Drug Administration (FDA), 14% of transfusion related fatalities between 2011 and 2015 were due to non-ABO hemolytic transfusion reactions [7]. Non-hemolytic transfusion reactions can be enormously challenging to prevent, particularly in cases when multiple or rare antibodies are involved. The risk of hemolytic transfusion reactions triggered by antibodies to low frequency antigens is relatively low, estimated to be around 1 per 650,000 [8]: the effects can nonetheless be devastating. Delayed hemolytic transfusion reactions have been studied extensively in the setting of sickle cell disease and beta-thalassemia [9–12], emphasizing the importance of understanding the risk of adverse events in patients with baseline hematologic deficiencies.

**Fig. 2:**
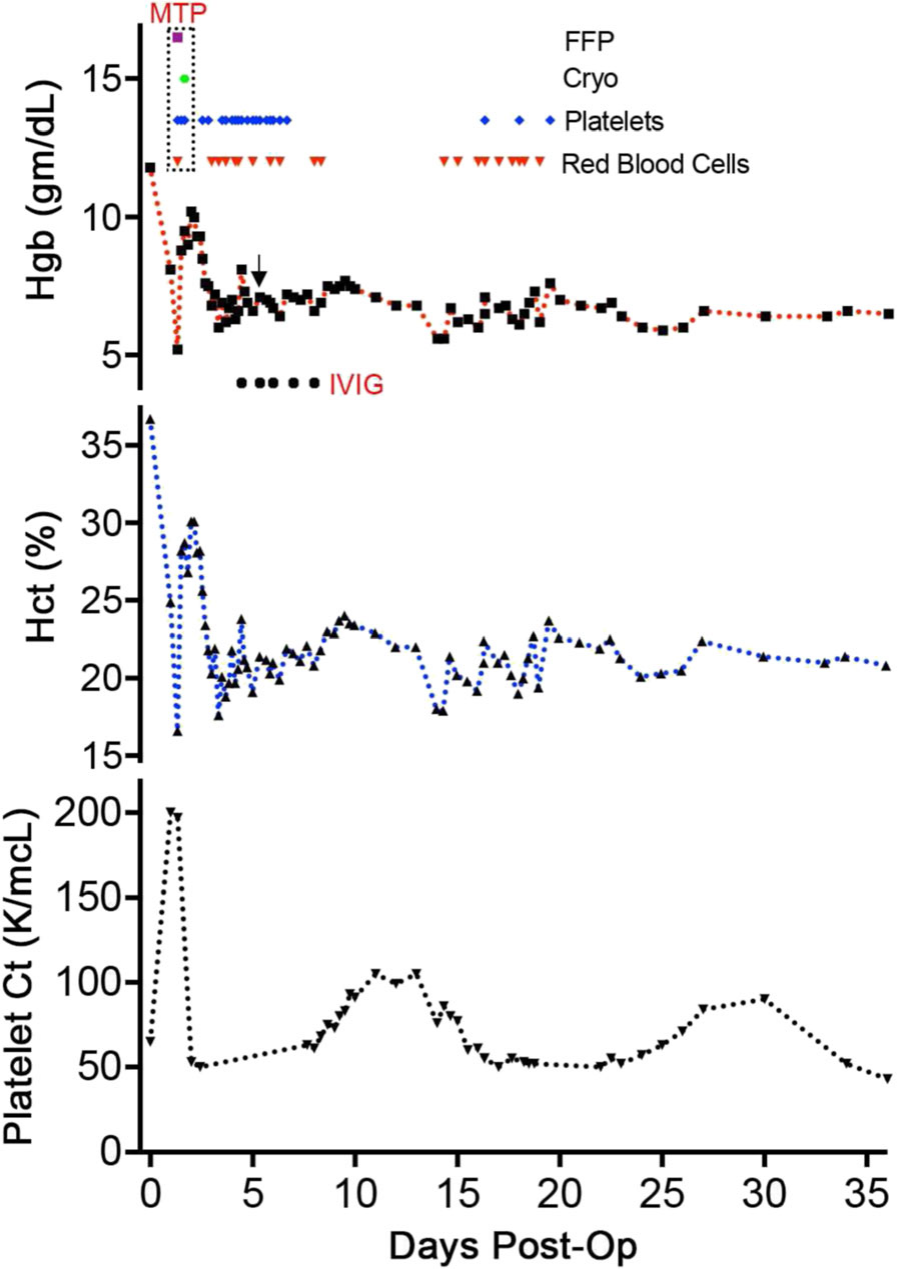
Shown are hemoglobin (Hgb), hematocrit (Hct) and platelet counts (Platelet Ct) over time (days). Additionally, transfusion of fresh frozen plasma (FFP – purple square), cryoprecipitate (Cryo – green circle), platelets (blue diamonds) and packed red blood cells (Red Blood Cells – red triangle) is indicated. Five administered doses of IVIG are indicated. Zero on the x-axis correlates with y-values prior to the initial hernia repair. The arrow indicates embolization of the spleen. MTP = massive transfusion protocol; IVIG = Intravenous immunoglobulin

Transfusion of red blood cells may trigger production of allo-antibodies capable of lysing incompatible donor RBCs, and in some cases the patient’s own blood cells as well [10]. In the case of DHTRs, lysis of RBCs generally does not occur until a few days after the initial transfusion of blood products. These may be caused by evanescent allo-antibodies in the setting of re-exposure to foreign antigens, or by new allo-antibody development to one of many known antigens. Hemolytic transfusion reactions have been reported to be caused by antibodies with varying specificities, including anti-c, anti-E, anti-Fy3, anti-Fy^a^, anti-Fy^b^, anti-Jk^a^, anti-Jk^b^, anti-K, anti-Kp^a^, anti-M, anti-N, anti-s and anti-U antibodies [13, 14]. Intravenous immune globulin (IVIG) associated hemolysis, used for the treatment of the patient presented below, has also been reported [15]. The symptoms may be somewhat nebulous and range from fever of unknown origin, to mild jaundice, to fatigue and to other non-specific findings [14]. A positive direct antiglobulin test (DAT) is telling; however, DAT can be negative in as many as 50% of cases [11]. Absence of the expected response to transfused blood products, as was seen in this case report (Fig. 2), can be a particularly important observation and should prompt withholding additional blood products [4, 10, 11].

TRALI is the leading cause of transfusion-related fatalities and is responsible for 38% of such fatalities [7]. Clinically, TRALI resembles acute respiratory distress syndrome (ARDS) – the underlying pathogenic mechanisms are however essentially different – and is associated with diffuse pulmonary edema, hypoxemia and hypotension [16]. The pathophysiology of TRALI is complex and incompletely understood; although, mouse models have contributed to our understanding of the disease mechanism [5, 17, 18]. TRALI can be either antibody-mediated (anti-human leucocyte antigen (HLA) class I or II or anti-human neutrophil antigen (HNA) antibodies) or non-antibody mediated [19]. A two-hit model is assumed to underlie the disease, in which the first hit is a patient predisposing factor, such as inflammation, while the second hit is present in the transfused product in the form of antibodies or biological response modifiers [5, 6, 20].

No definitive diagnostic test exists for TRALI to date and its diagnosis remains clinical. The US National Heart, Lung and Blood Institute Working Group and a consensus panel have formulated a definition for TRALI based on radiological and clinical criteria [6, 21–23]:

1. *Acute onset of respiratory distress within 6 h of blood transfusion*
2. *PaO*_*2*_ */FiO*_*2*_ *ratio of <300 mmHg or worsening PaO*_*2*_ */FiO*_*2*_ *ratio*
3. *Newly developed or worsened bilateral pulmonary infiltrates indicative of pulmonary edema on chest X-ray*
4. *No signs of hydrostatic pulmonary edema or cardiac ischemia and transfusion associated circulatory overload (TACO)*
5. *No other risk factors for acute lung injury (ALI)*

“Possible” TRALI is defined using the same criteria used for TRALI, but also includes the presence of a clear temporal association for an alternative risk factor for ALI [5, 24].

Known risk factors for TRALI are: liver transplantation surgery, chronic alcohol abuse, shock, high peak airway pressure with mechanical ventilation, current smoker, pneumonia, sepsis, multiple fractures, pancreatitis, aspiration and positive fluid balance [24, 25]. In addition, inflammation is also a major risk factor for TRALI and is characterized by elevated interleukin (IL)-6, IL-8 [25–27] and elevated C-reactive protein (CRP) levels [18, 28].

Enormous efforts over the past decade have led to a significant reduction in TRALI, driven by the introduction of low risk plasma (male plasma for transfusion and HLA antibody screening of female donors; [29]). Studies have shown that blood products form multiparous donors has the greatest probability of triggering TRALI [3, 7, 29]. However, as indicated by the mortality rate associated with TRALI, and given the lack of full understanding of the underlying pathophysiology, it should be evident that this potential transfusion-associated complication cannot be dismissed and suspicion must be high, particularly when a massive transfusion protocol is activated.

## Case presentation

**Fig. 3:**
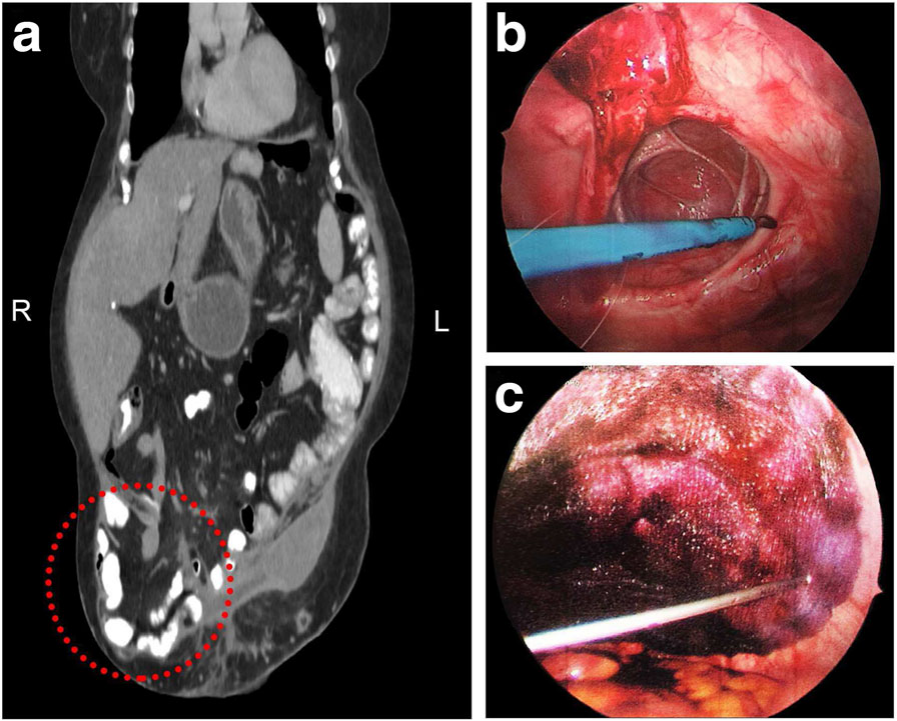
**a** Coronal view of the computerized tomography (CT) scan. Laparoscopic view of a 4-cm ventral hernia before **b** and after **c** closure with Ventrio ST mesh. Red ellipse = hernia; R = right; L = left

The patient is a 53-year-old woman (Fig. 1) with a complicated medical history including cirrhosis, non-alcoholic fatty liver disease, morbid obesity and immune thrombocytopenia (ITP) (Fig. 2; a platelet count of less than 100,000/microliter at post-operative day zero). Her past surgical history is significant for gastric bypass with Roux-en-Y for morbid obesity, hernia repairs, hysterectomy and tubal ligation. The patient presented for repair of her symptomatic ventral hernia (Fig. 3). Surgical repair of the hernia was performed laparoscopically (Fig. 3) and was achieved without apparent complications. The patient was extubated after surgery and moved to recovery.

Overnight, the patient complained of significant abdominal pain and tenderness and abdominal ecchymoses were noted in addition to concerning hypotension with systolic blood pressures of 60-70 mmHg and diastolic blood pressures of 40-50 mmHg. The patient was taken to the operating room emergently for exploratory laparotomy, which reveled acute hemoperitoneum. All blood was aspirated using Cell Saver. Careful inspection of the repaired hernia did not suggest it as the site of bleeding; the liver also did not appear to be damaged. Minor oozing of blood from the omentum was noted as the only site of active bleeding: prompting a partial omentectomy. The patient’s baseline thrombocytopenia (Fig. 2), is the likely reason for continuous bleeding. Coagulation tests done on post-operative day one revealed a high partial thromboplastin time (PTT) of 36.9 s and low levels of fibrinogen (140 mg/dL). The international normalized ratio (INR) was normal at 1.1. Post-operatively, the patient remained intubated and was transferred to the surgical intensive care unit (SICU) for close observation. A massive transfusion protocol (MTP) was activated in the SICU, where within 24 h the patient received three units of platelets (one additional unit had been given in the operating room), three units of fresh frozen plasma, four units of packed red blood cells and ten units of cryoprecipitate. This measure was triggered by the patient’s drop in hemoglobin and hematocrit from pre-operative levels of 11.8 gm/dL and 36.7% to 5.2 gm/dL and 16.6%, respectively (Fig. 2). Possible massive transfusion-related hemolysis was considered at this point; however, pre- and post-transfusion DAT were negative. The patient was positive for anti-E and anti-K antibodies, which was known from previous testing. Accordingly, all transfusions were phenotypically matched. Within 4 h of activating the MTP (Fig. 1), the patient’s respiratory condition deteriorated, necessitating positive end-expiratory pressure (PEEP) of 14 with a fraction of inspired oxygen (FiO_2_) level of 100%.

**Fig. 4:**
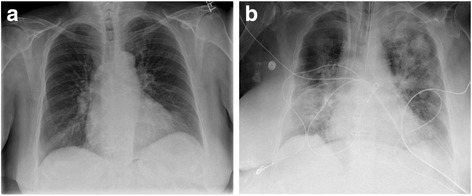
Pre-transfusion **a** and post-transfusion **b** chest x-rays

The patient’s hemoglobin and hematocrit dropped precipitously following a brief increase after the massive transfusion. Moreover, the patient’s platelet count quickly returned to a level comparable to the pre-transfusion level, 50 K/mcL (Fig. 2), which is only slightly below to the patient’s baseline (70-90 K/mcL). Due to the patient’s continuously low hemoglobin and hematocrit, relatively steady at 6-7 gm/dL and 19-21%, respectively, additional blood products were transfused: all with minimal apparent impact (Fig. 2). The patient remained intubated and a post-operative chest X-ray four hours after the first transfusion of blood products showed mild to moderate reduction in lung volume and moderate diffuse airspace disease bilaterally; heart and mediastinum were within normal limits (Fig. 4).

Based on the patient’s risk factors (high peak airway pressure with mechanical ventilation, possible shock and a positive fluid balance), the bilateral infiltrates on chest radiograph without evidence of left atrial hypertension and the significant persistent respiratory distress shortly after transfusion of blood products, a diagnosis of TRALI was made. CRP levels [18, 28] and interleukin levels [25–27] were not evaluated, two parameters of potential value for the assessment of TRALI.

Review of donor histories from the transfused products revealed one female donor with a pregnancy history [29]. Subsequent screening of the donor identified positive HLA class I and class II antibodies, strongly raising the possibility of “true” antibody-mediated TRALI [5, 6, 30]. The donor refused additional testing to confirm a match with the patient’s HLA type.

**Fig. 5:**
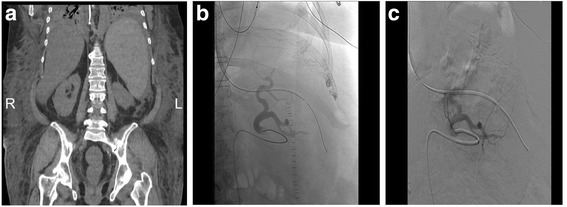
**a** Coronal views of the patient’s enlarged spleen on CT scan. Arteriogram of the upper pole branch of the splenic artery, performed with CO_2_ gas followed by Visipaque contrast, pre- **b** and post-embolization **c** Gelfoam mixed with gentamicin was used to achieve complete stasis

Despite the frequent transfusion of blood products, the patient’s hematologic status remained unstable (Fig. 2). Given the patient’s critical status, interventional radiology was consulted and performed Gelfoam embolization of the upper pole branch of the splenic artery, to treat the patient’s hypersplenism and severe thrombocytopenia (Fig. 5). Additionally, 5 doses of IVIG were administered to treat the thrombocytopenia – several reports describe that IVIGs were administered to treat ITP [31–33] – also with very limited impact (Fig. 2).

Even though initial pre- and post-operative DATs had been negative, on post-operative day 16 laboratory technologists noticed that the patient’s serum had changed in appearance from icteric to hemolytic (Fig. 1). Follow-up laboratory studies indeed indicated in vivo hemolysis: lactate dehydrogenase (LDH) levels of 1,180 IU/L, haptoglobin of less than 5.8 mg/dL and a reticulocyte count of 4.38% (Fig. 1). Total (2.1 mg/dL) and direct bilirubin (1.3 mg/dL) were marginally elevated. An extensive blood bank work-up was initiated at this point; antibody screening was positive, and, as expected, the anti-K and anti- E antibodies were identified. Serial DATs now began to show positive results. Several panels were run, but an additional known antibody could not be identified. Units that had previously tested as compatible were tested with newer specimens and were now incompatible. Based on these findings all transfusions were immediately halted and no additional blood products were given for the remainder of the patient’s hospitalization. A rare allo-antibody was suspected, and samples were sent to the Blood Centers of Wisconsin. Given the positive repeat DAT, “super-coombs” testing was unnecessary. At the reference center, extensive testing ruled out known uncommon antibodies, including: Vel, Cs(a), Yk(a), Cr(a), Lu-8, Yta, AnWj, Lua-b-, Ata, Ko, Lw(a-b+), Kna, McC(a), Sl(a), Ch, Ge-2, -3, Ge-2, In(b-), Tc(a-), Jr(a-), U-, Sc:-1, Ena-, Wrb, Lan. Overall, these blood bank findings are consistent with unknown, rare allo-antibody formation to a red blood cell antigen.

The patient’s hemoglobin, hematocrit and platelet levels continued to be low throughout the post-operative period, but were very stable with the administration of iron and epogen (Fig. 2). The patient started to improve significantly as management was adjusted, including stringent restrictions of all transfusions. Laboratory values and vital signs started to normalize: LDH 513 IU/L, direct bilirubin 0.2 mg/dL, haptoglobin 51.7 mg/dL (Fig. 1), and the patient’s blood pressure returned to her pre-operative levels of 120-130/60 mmHg with a heart rate of 80 beats per minute. Importantly, the patient started to tolerate trach collar and a regular diet and was quickly able to ambulate again. Subsequently, the patient was transferred to a rehabilitation center and appeared to be regaining her health. The slow and cumbersome healing process and the significant setbacks were extremely frustrating for the patient. During the worst part of her recovery, the patient felt quite emotionally drained and expressed feelings of deep depression. All possible complications had been discussed at length prior to surgery; nonetheless, the severity of her complications was unexpected, particularly following a routine hernia repair. Addressing the patient’s psychological needs was another critical step that had to be taken as the patient’s care was optimized.

## Conclusions

Hemolytic transfusion reactions and TRALI are life-threatening adverse events associated with massive transfusion of blood products [7]. In specific patient populations (i.e., with multiple or rare allo-antibodies and evidence of TRALI), once active bleeding has been controlled, a high threshold for administering additional transfusions should be maintained. The recommended management of patients like the one described in this report focuses on having a high index of suspicion even pre-operation, involving the blood bank early, performing early testing and withholding transfusions as soon as possible. Despite great need, no specific therapies for TRALI are currently available, apart from supportive measures such as oxygen and ventilation.

## Abbreviations

ALI: Acute lung injury
Cryo: Cryoprecipitate
CT: Computerized tomography
CXR: Chest x-ray
DAT: Direct antiglobulin test
DHTR: Delayed hemolytic transfusion reaction
FDA: Food and Drug Administration
FFP: Fresh frozen plasma
FiO_2_: Fraction of inspired oxygen
Hct: Hematocrit
Hgb: Hemoglobin
HLA: Human leucocyte antigen
HNA: Human neutrophil antigen
INR: International normalized ratio
ITP: Immune thrombocytopenia
IVIG: Intravenous immunoglobulin
LDH: Lactate dehydrogenase
MTP: Massive transfusion protocol
PaO_2_: Arterial partial pressure of oxygen
PEEP: Positive end-expiratory pressure
PEG: Polyethylene glycol
Platelet Ct: Platelet counts
PTT: Partial thromboplastin time
RBC: Red blood cells
SCD: Sickle cell disease
SICU: Surgical intensive care unit
TACO: Transfusion associated circulatory overload
TRALI: Transfusion-related acute lung injury
